# Nematicidal effect of cruciferous bio-fumigants against the root-knot nematode, *Meloidogyne incognita* infesting okra

**DOI:** 10.21307/jofnem-2020-080

**Published:** 2020-07-21

**Authors:** J.A. Patil, Anil Kumar, Saroj Yadav, K.K. Verma

**Affiliations:** Department of Nematology, College of Agriculture, CCSHAU Hisar, Hissar, 125004 Haryana, India

**Keywords:** Bio-fumigation, Cabbage leaves, carbofuran, Cauliflower leaves

## Abstract

In Haryana, India, only carbofuran is registered for the management of root-knot nematodes. The objective of this study was to investigate the potential of cruciferous bio-fumigants for the management of root-knot nematodes (*Meloidogyne incognita*) in okra. The experiments were conducted at research area Department of Nematology in 2017 to 2018 and 2018 to 2019. During this investigation, cruciferous bio-fumigants such as cabbage leaves and cauliflower leaves were used as bio-fumigant sources to protect Okra cv. Hisar Unnat. Fresh and chopped leaf mass of cabbage and cauliflower was incorporated uniformly into a naturally infested field. The initial nematode population in both years was 224 and 256 J_2_/200 cc soil, respectively. The results of our investigation showed that in both the years okra yield was enhanced significantly by the measures of nematode management. In addition, both of the tested bio-fumigant plants leaves proved to be potentially promising for the management of root-knot nematodes. Among the bio-fumigants, the highest decrease in nematode population, root gall index and increase in yield was observed in cabbage leaves @ 50 t/ha in both years, consecutively.

Okra (*Abelmoschus esculentus* L. Moench) is a biannual crop with two growing seasons, starting in early March and late July. Based on its rich nutritional contents, this vegetable is very important in India, even though it was originated from the tropics of Afro-Asian countries. It is rich in protein, and the predominant elements found in this vegetable are K, Mg, Na, Ca, and Fe (Santos et al., 2013). It also contains vitamins A and B (Gemede et al., 2014). Production of okra is strongly influenced due to the attack by several pathogens, such as bacteria, fungi, viruses, nematodes, and abiotic factors. Plant parasitic nematodes are the most harmful pests of vegetable crops, responsible for an annual yield loss amounting to 9 to 15% of the world crop yield (Koenning et al., 2004). Among all the plant parasitic nematodes, root-knot nematodes (*Meloidogyne* spp.) are a hidden threat to okra (Marin et al., 2017). It has been reported that root-knot nematode causes annual losses up to 29% in tomato, 22% in okra, 24% in potato, 23% in eggplant, 25% in pepper, and 28% in beans (Sasser, 1979). Currently, different strategies are used for minimizing nematode populations such as crop rotation and use of non-host crops (Ahmad et al., 2004; Hussain et al., 2011). Moreover, the use of soil-applied nematicides, most of which are relatively ineffective (Mukhtar et al., 2013), also has the risk to human health and environment. Therefore, economically viable and sustainable approaches are required for the management of root-knot nematodes. Ergo, these management strategies are limited because there is no any resistant variety available against root-knot nematode in okra in Haryana, India, and the wide host range of this nematode makes it difficult to design a practical and effective cropping system. Cruciferous bio-fumigants have been proposed as another strategy to manage soil-borne problems including nematodes (Stirling and Stirling, 2003; Matthiessen and Kirkegaard, 2006).

Bio-fumigation is very important agronomic practice of using volatile chemicals (allelochemicals) released from plant tissues after the decomposition to suppress pests (Kumar and Mishra, 2014). The term bio-fumigation was coined by different scientists to describe the suppression of soil-borne pathogens by releasing different compounds by brassica species (Motisi et al., 2010). Bio-fumigation has been widely researched by many countries, namely Australia, Italy, South Africa, New Zealand, the Netherlands and the USA. Asian countries like Cambodia, China, India, Thailand, and Philippines have also been involved in bio-fumigation trials (Kumar, 2005). Bio-fumigation has a lot of potential in Asian countries because cruciferous crops are widely grown and consumed. This strategy is an alternative to the environmentally damaging chemical fumigants and sterilants (Gouws, 2004). Therefore, bio-fumigation techniques are developing worldwide. It has been shown that there is a potential to use bio-fumigation as an alternative to methyl bromide in horticulture and broad agriculture to manage pests (Ghoname et al., 2015). Majority of the studies on bio-fumigation have been conducted in laboratory and greenhouse conditions. Keeping all the facts in mind, this study was performed to evaluate the efficacy of cruciferous bio-fumigants against root-knot nematode, *Meloidogyne incognita* infesting okra under field conditions in 2017 to 2018 and 2018 to 2019.

## Materials and methods

The experiment was conducted in years 2017 to 2018 and 2018 to 2019 in research area, Department of Nematology, CCSHAU Hisar, Haryana, India (Latitude: 29°10´N, Longitude: 75°46´E, and Altitude: 215.2 m), which has subtropical and tropical climate with an annual average temperature of 25.1°C and an average annual rainfall of 459 mm.

### Field preparation

Root-knot nematode inoculum was obtained from okra infested field of Hisar, Haryana, India. It was maintained and cultured on tomato variety ‘Hisar arun’ and species identified by using the perineal pattern (Netscher and Taylor, 1974). The field trial was conducted at the CCS Haryana Agricultural University research area, Department of Nematology. Soil type was a sandy loam having sand 89.7%, silt 4.5%, clay 5.8%, organic matter 1%, and pH 7.2. Before conducting this experiment, the field was sterilized with formalin followed by soil solarization. Then nematode infestation was maintained for seven years by growing different susceptible crops like okra (*Abelmoschus esculentus*), eggplant (*Solanum melongena*), and tomato (*Solanum lycopersicum*) for seven years.

### Care and maintenance of plants

The plants were observed regularly, and watering, hoeing, etc., were done whenever required. Fertilizers were applied according to the recommended dosage for okra.

### Experimental procedure

The effect of two cruciferous species, namely cauliflower (*Brassica oleracea* var. botrytis) and cabbage (*Brassica oleracea* var. capitata), was evaluated over two years. Plot size was 3.0 × 2.1 m. The initial nematode population (INP) in the soil before the incorporation of leaves was 224 J_2_/200 cc and 256 J_2_/200 cc in 2017 to 2018 and 2018 to 2019, respectively. Fresh and chopped leaf mass of cruciferous bio-fumigants was incorporated uniformly into infested field, 15 days before sowing. After 15 days of incorporation of leaves, nematode populations were counted from treated plots (Tables 1 and 2). The okra variety used in the experimentation was Hisar Unnat. The experiment was designed according to randomized block design (RBD) with six treatments − T1: cabbage leaves @ 25 t/ha or 2.5 kg/m^2^, T2: cabbage leaves @ 50 t/ha or 5 kg/m^2^, T3: cauliflower leaves @ 25 t/ha or 2.5 kg/m^2^, T4: cauliflower leaves @50 t/ha or 5 kg/m^2^, T5: carbofuran @ 1.0 kg a.i./ha, and T6: untreated control and treatments were replicated four times.

**Table 1.** Management of root-knot nematode in okra by bio-fumigation (2017-2018).

Sr. No.	Treatments	INP/200 cc soil before addition of leaves	INP/200 cc soil after addition of leaves	FNP/200 cc soil at harvest	Okra yield (kg/plot)	% increase in yield/over check	Root-knot index at harvest
1	Cabbage leaves @ 25 t/ha	224.0	197.0	258.0^b,c^	8.6^b,c^	8.9	3.8^a,b^
2	Cabbage leaves @ 50 t/ha	224.0	185.0	215.7^d,e^	9.9a	25.3	3.3^a^
3	Cauliflower leaves @ 25 t/ha	224.0	203.0	268.0^b^	8.2^c,d^	3.8	4.0^a,b^
4	Cauliflower leaves 50 t/ha	224.0	200.0	231.0^d^	9.2^a,b^	16.4	3.5^a^
5	Carbofuran @ 1.0 kg a.i./ha	224.0	153.0	188.0^f^	8.4^c,d^	6.3	3.5^a^
6	Untreated check	224.0	215.0	340.6^a^	7.9^c,d^	−	5.0^c^
7	CD @ 5%	−	−	19.8	0.8	−	0.83

**Notes:** INP, initial nematode population; FNP, final nematode population. All treatments had four replications in a randomized block design. Values with same letters in column denote non-significant difference according to critical difference (CD).

**Table 2.** Management of root-knot nematode in okra by bio-fumigation (2018-2019).

Sr. No.	Treatments	INP/200 cc soil before addition of leaves	INP/200 cc soil after addition of leaves	FNP/200 cc soil at harvest	Okra yield (q/ha	% increase in yield/over check	Root-knot index at harvest
1	Cabbage leaves @ 25 t/ha	256	225	274^e^ (15.60)	65.5^c^	14.0	2.8^a,b^
2	Cabbage leaves @ 50 t/ha	256	151	226^f^ (15.05)	78.2^a^	36.2	2.5^a^
3	Cauliflower leaves @ 25 t/ha	256	242	290^c^ (17.06)	63.2^c,d^	11.2	3.3^a,b^
4	Cauliflower leaves 50 t/ha	256	181	268^d^ (16.40)	71.8b	25.0	3.0^a,b^
5	Carbofuran @ 1.0 kg a.i./ha	256	194	250^a^ (20.83)	63.4^c,d^	11.2	3.5^b^
6	Untreated check	256	265	417^b^ (20.43)	57.1e	−	4.8^c^
7	CD @ 5%	−	−	(0.13)	5.1		0.89

**Notes:** INP, initial nematode population; FNP, final nematode population. Figures in parenthesis are √ transformed values. All treatments had four replications in a randomized block design. Values with different letters in column denote significant difference according to critical difference (CD).

#### Data collection

Data were calculated for okra yield expressed as q per ha and nematode reproduction parameters (INP/200 cc soil before the addition of cabbage/cauliflower leaves, INP/200 cc soil at the time of sowing of okra, final nematode population/200 cc soil, root-knot index at harvest). The percentage increase and decrease in the yield over the control were calculated by the following formula:

$$\;\%\mathrm{Decreaseorincreaseinyield}=\frac{\mathrm{Mean}\;\mathrm{yield}\;\mathrm{of}\;\mathrm{treated}\;\mathrm{plot}\;–\;\mathrm{Mean}\;\mathrm{yield}\;\mathrm{of}\;\mathrm{untreated}\;\mathrm{plot}\;}{\mathrm{Mean}\;\mathrm{yield}\;\mathrm{of}\;\mathrm{untreated}\;\mathrm{plot}}\times 100$$

Roots from these plants were indexed for galling and egg mass presence on a scale from 1 to 5 where 1.0 = no galls or egg masses, 2.0 = 1-10 galls or egg masses, 3.0 = 11-30, 4.0 = 31-100 galls or egg masses, and 5.0 = more than 100 galls or egg masses (Heald et al., 1989). For the estimation of nematode populations, soil was processed as per the sieving method of Cobb’s sieving and decanting technique (Cobb, 1918) followed by the Modified Baermann’s funnel technique (Viglierchio and Schmitt, 1983). The extracted second-stage *M. incognita* juveniles (J_2_) were counted at 40 × magnification.

#### Statistical analysis

The data were subjected to RBD using OPSTAT program available online at CCSHAU Hisar University website (www.hau.ernet.in). The comparisons in treatments were made by critical difference at the 5% level of significance. Necessary transformations of data were done where applicable. The relationships between the number of galls and the yield were determined using regression analysis with Excel 2016.

## Results

### Results achieved during 2017 to 2018

**Figure 1:**
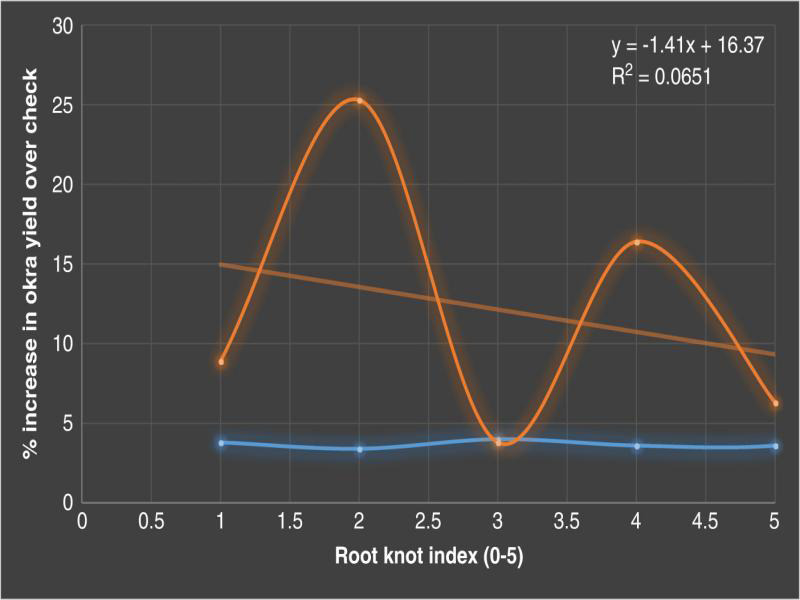
Relationship between number of galls and percentage increase in okra yield over check (2017-2018).

The multiplication of *M. incognita* in plots treated with cruciferous bio-fumigants was significantly lower than the treatments with carbofuran. Finally, cabbage leaves resulted to be significantly more suppressive than cauliflower leaves. Treatments with the four cruciferous bio-fumigants and carbofuran also resulted in a lower number of root galls in comparison with untreated check. As for nematode juveniles, the formation of galls in soil treated with cruciferous bio-fumigants and carbofuran was lower than untreated check. However, the results (Table 1) indicated that okra yield was significantly highest in the treatment of cabbage leaves @ 50 t/ha followed by cauliflower leaves @ 50 t/ha. The yield in these two treatments was even better than chemical check, i.e., carbofuran @ 1.0 kg a.i./ha. There was 25.3% increase in yield over the check in cabbage leaves @ 50 t/ha followed by 16.4% in cauliflower leaves @ 50 t/ha. Therefore, the final nematode population in the soil as well in roots was lowest in carbofuran @ 1.0 kg a.i./ha (188 J_2_/200 cc soil) followed by cabbage leaves @ 50 t/ha (215 J_2_/200 cc soil). The regression studies showed (Fig. 1) negative and significant relationship between number of galls and percentage increase in okra yield over the check (*r*² = 0.0651).

### Results achieved during 2018 to 2019

**Figure 2:**
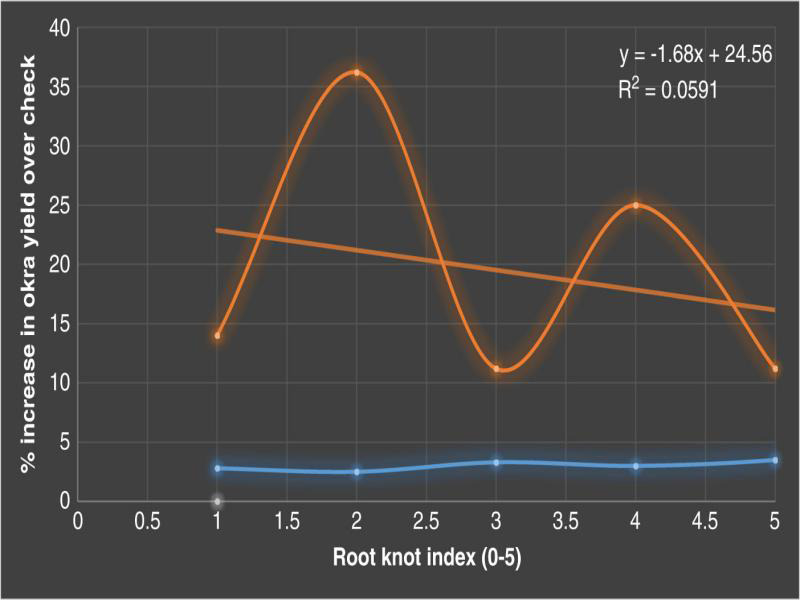
Relationship between number of galls and percentage increase in okra yield over check (2018-2019).

After the incorporation of cruciferous bio-fumigants, the highest decrease in nematode juvenile population was observed in cabbage leaves @ 50 t/ha (151 J_2_/200 cc soil) followed by cauliflower leaves @ 50 t/ha (181 J_2_/200 cc soil). A highest reduction in the gall index of okra roots was obtained from field plots incorporated with cruciferous bio-fumigants but not in untreated check (Table 2). The carbofuran-treated plots also had significantly lower galling compared with untreated check. Gall indices were detected 2.5 and 3.0 for roots of okra in plots treated with cabbage leaves @ 50 t/ha and cauliflower leaves @ 50 t/ha, respectively. Positive effect of cabbage leaves @ 50 t/ha was increased 36.2% in yield over check followed by 25.0% in cauliflower leaves @ 50 t/ha. Okra yields obtained from the plots treated with cruciferous bio-fumigants and the carbofuran were all significantly higher than that of untreated check (Table 2). The final nematode population in the soil was lowest in cabbage leaves @ 50 t/ha (226) followed by carbofuran @ 1.0 kg a.i./ha (250). The regression studies showed (Fig. 2) negative and significant relationship between number of galls and percentage increase in okra yield over check (*r*² = 0.0591).

## Discussion

In recent years, bio-fumigation has emerged as an effective non-chemical alternative to manage nematode pests. However, these results aim to be only indicative of the potential use of cruciferous bio-fumigants for nematode management and need to be validated by future trials in farmers field conditions, as well as different combinations of bio-agents with cruciferous bio-fumigants should also be tested to verify a potential synergism among different practices. The experimental study indicated that cruciferous bio-fumigants have provided a satisfactory nematode suppression, confirming previous findings of some researchers (Haroutunian, 2013). In cruciferous bio-fumigants, the enzyme (myrosinase)-catalyzed hydrolysis of sulphur-containing substrate (glucosinolate) initially involves cleavage of the thioglucoside linkage, yielding D-glucose and an unstable thiohydroximate-O-sulphonate, which spontaneously rearranges to produce sulphate and a number of reaction products (Zhou et al., 2012). The end products are generally isothiocynate, thiocyanate, nitrile, epithionitrile, or oxazolidine-thione, depending on substrate, pH, or ferrous ions available (Zhou et al., 2012; Avato et al., 2013). In field environment, both fresh chopped cruciferous bio-fumigants had significantly inhibited the galling of *M. incognita* in okra. Our findings are in agreement with Valdes et al. (2012) that show cruciferous bio-fumigants not only reduce galling but are also responsible for the increase in beneficial nematode community in soil profile which is also reported due to brassica bio-fumigation effect. Studies on bio-fumigation have been done with brassicas for the management of nematodes in different cropping systems. (Kumar, 2005; Matthiessen and Kirkegaard, 2006; Henderson et al., 2009; Szczygłowska et al., 2011). After the incorporation of cruciferous bio-fumigants into the field, significant reduction in the population of nematode juveniles occurred. Researchers already have proved that after the decomposition of biofumigant, plant tissues mainly releases isothiocyanates, in addition to thiocyanates, nitriles, and oxazolidinethiones (Fahey et al., 2001; Devi, 2018). GCs are sulphur-containing chemicals that are produced by plants as secondary metabolites (Agrios, 2005). Nematode reproduction was reduced in the treated plots with cruciferous bio-fumigants as compared with chemical check. Similar finding were reported by Bello et al. (2001) who showed that bio-fumigation has the same effect as methyl bromide on *Meloidogyne javanica* in pepper. As reported by many scientists, field experiments in which *Brassica juncea* and *Brassica rapa* were grown as green manure crops resulted in 91 to 95% mortality of encysted eggs of *Globodera pallida* (Lord et al., 2011). On the basis of our finding, we suggest to farmers in root-knot nematode infested field first grow the cruciferous crops in Winter and after that okra in Summer. Brassicas are already known to be tolerant to root-knot nematodes (Bünte et al., 1997; Page, 1997), and they are included as rotational crops in cropping programs that contain susceptible vegetable crops like tomatoes, paprika, and peas.

## Conclusion

Most used nematicides banned from world markets and trends toward natural farming and sustainable agriculture continue to escalate; more research and outreach effort are necessitated towards developing and disseminating information on alternative PPN management strategies. One such strategy is the use of cruciferous bio-fumigants against root-knot nematode. Although a plethora of research have been conducted on using these crops for root-knot nematode management, agricultural stakeholders remain to be convinced to integrate bio-fumigation into their INM practices. Indeed, it is unlikely that bio-fumigation as a standalone technique will eliminate target PPNs in soil, but this technique can easily be integrated with other strategies such as soil solarization, minimal use of nematicides, use of resistant varieties, bio-control agents, etc., in a cost-effective manner to provide acceptable levels of nematode management.
